# Eating patterns in a nationwide sample of Japanese aged 1–79 years from MINNADE study: eating frequency, clock time for eating, time spent on eating and variability of eating patterns

**DOI:** 10.1017/S1368980021000975

**Published:** 2022-06

**Authors:** Kentaro Murakami, M Barbara E Livingstone, Shizuko Masayasu, Satoshi Sasaki

**Affiliations:** 1 Department of Social and Preventive Epidemiology, School of Public Health, University of Tokyo, Tokyo 113-0033, Japan; 2 Nutrition Innovation Centre for Food and Health (NICHE), School of Biomedical Sciences, Ulster University, Coleraine, UK; 3 Ikurien-naka, Ibaraki, Japan

**Keywords:** Meals, Snacks, Eating occasions, Dietary record, Eating behaviours

## Abstract

**Objective:**

Although there is growing evidence suggesting that eating patterns are important determinants of health status, comprehensive information on patterning of eating behaviours is almost lacking. The aim of this cross-sectional study was to describe eating patterns in Japan.

**Design:**

Information on actual eating behaviours was collected using 2-d dietary record in each season over a year (total 8 d). Eating occasions were defined as any discrete intake occasion (with a discrete start clock time and name) except for eating occasions consisting of water only, which were excluded.

**Setting:**

Japan.

**Participants:**

A nationwide sample of 4032 Japanese aged 1–79 years.

**Results:**

The mean value of eating frequency of meals (i.e. breakfast, lunch and dinner), snacks and total eating occasions was 2·94, 1·74 and 4·68 times/d, respectively. The mean clock time for the start of breakfast, lunch and dinner was 07.24, 12.29 and 19.15 h, respectively. The mean time spent consuming breakfast, lunch, dinner and snacks was 19, 25, 34 and 27 min/d, respectively. On average, variability (i.e. average of absolute difference from mean) of meal frequency was small compared with that of snack frequency and total eating frequency. Both mean variability of clock time for the start of eating (<1 h) and mean variability of time spent on meals (<10 min/d) were also small. Conversely, mean variability of time spent on snacks was large (>18 min/d).

**Conclusion:**

The present findings serve as both a reference and an indication for future research on patterning of eating behaviours.

Efforts to overcome the limitations of evaluating the health impact of consuming single nutrients and foods in isolation have led to a gradual shift in nutrition research to dietary patterns^(1,2)^. While dietary patterns are generally examined using the daily intake of individual foods^(3–6)^, an increasing number of studies now focus on dietary intake on an eating occasion basis (i.e. breakfast, lunch, dinner and snacks) or eating patterns^(7–9)^. Evaluation of eating patterns instead of overall dietary intakes or patterns might increase relevance by accounting for physiological synergies and interactions occurring during digestion and metabolism^(10)^. Moreover, eating pattern-based dietary advice would resonate better with consumers if it reflects actual eating behaviours^(9)^.

Because of the complex nature of eating patterns, existing research has used a variety of variables in terms of eating patterns, including eating frequency^(11–17)^, timing of eating^(13,18–21)^ and variability of eating patterns^(22–27)^, with equivocal outcomes. This may be mainly due to a lack of clear definitions of these variables. To develop more consistent and clearer definitions of eating pattern variables, comprehensive reports on eating pattern variables based on actual eating behaviour assessment (such as dietary record) are imperative. While there are several papers in this regard based on large-scale observational studies in free-living situations^(11–14,18)^, these are limited with regard to the number of dietary assessment days (usually only 1 or 2 d)^(11–13,18)^ or assessed only a limited aspect of eating patterns^(11,14,18)^. In particular, we are unaware of descriptive information on time spent on eating and variability of eating patterns in general populations despite the potential health effects of eating rate^(28,29)^ and regularity of eating^(22–26)^. Moreover, most of previous studies have been conducted in adult populations, while information in children is sparse. Investigation of this issue in children is merited from a prevention perspective.

Therefore, the aim of the present study was to describe eating patterns, namely eating frequency, clock time for the start of eating, time spent on eating and variability of eating patterns, in a nationwide sample of Japanese aged 1–79 years, based on information on actual eating behaviors collected using 2-d dietary record in each season over a year (total 8 d).

## Methods

### Study procedure and participants

This analysis was based on data from MINNADE (MINistry of health, labour and welfare-sponsored NAtionwide study on Dietary intake Evaluation) study. The ultimate purpose of MINNADE study was to describe nationwide data on dietary characteristics and eating behaviours in Japan. The study consisted of two rounds of 1-year data collection (first round: November 2016 to September 2017; second round: October 2017 to September 2018). The target population comprised apparently healthy Japanese aged 1–79 years living in private households in Japan. Initially, thirty-two (of forty-seven) prefectures, which cover >85 % of total population in Japan, were selected on the basis of geographical diversity and feasibility of the survey, particularly the recruitment of collaborators (research dietitians). During sampling procedure, the proportion of population number in each region in Japan was reflected (i.e. Hokkaido 4 %, Tohoku 7 %, Kanto I 28 %, Kanto II 8 %, Hokuriku 4 %, Tokai 12 %, Kinki I 13 %, Kinki II 3 %, Chugoku 6 %, Shikoku 3 %, Kita-kyushu 7 % and Minami-kyushu 5 %^(30)^).

A total of 441 research dietitians agreed to support the study and were responsible for recruitment of participants from communities as well as data collection. Based on feasibility and human and financial resources (assuming 5–6 persons per research dietitian), we decided to include 256 individuals (128 for each sex) for each of nine age groups (i.e. 1–6, 7–13, 14–19, 20–29, 30–39, 40–49, 50–59, 60–69 and 70–79 years) during the first round of data collection (*n* 2304 in total). Considering the difference in dropout rate between age-sex groups observed during the first round, the number of recruited participants in the second round varied from 110 to 119 for each sex-age group, with a total of 2051 individuals (4–5 persons per research dietitian).

The key inclusion criterion for this study of community-dwelling (free-living) individuals was their willingness to complete a dietary record. Excluded from the study were dietitians, individuals living with a dietitian, those working together with a research dietitian, those who had experienced dietary counselling from a doctor or dietitian, those taking insulin treatment for diabetes, those taking dialysis treatment, pregnant or lactating women (at the start of study) and infants habitually drinking human milk. We did not exclude overnight workers from this study but asked not to conduct dietary record on overnight working days as well as days before and after these days. Participation of only 1 person per household was permitted. Participants in this study were not randomly selected. Consequently, a total of 4268 individuals aged 1–79 years participated in this study.

### Dietary assessment

Dietary data were collected using 4 × 2-d (total 8 d) weighed dietary records. After receiving written and verbal instructions by a research dietitian, as well as an example of a completed diary sheet, each participant was requested to maintain a record of all items eaten or drunk, both in and out of the home. This was done over 2 nonconsecutive days once per season at an interval of around 3 months, namely November for fall, February for winter, May for spring and August for summer. The sets of two recording days comprised 2 weekdays (Monday to Friday) for half of participants and 1 weekday and 1 weekend day (Saturday or Sunday as well as national holidays) for the remaining participants. However, not all days of the week were evenly represented. This allocation was maintained throughout the study; thus, it was expected that half of participants provide 8-weekday dietary data while the remaining participants provide 4-weekday data and 4-weekend day data. This strategy was adopted to obtain dietary data with an approximate proportion of weekdays and weekend days (3:1 compared with the actual ratio of 5:2) as a whole, while not compromising feasibility and simplicity for the conduct of the survey. The recording schedule for each participant was arranged by the assigned research dietitian.

Children aged ≥13 years (as well as adult participants) were expected to be able to complete the record themselves, whereas for children aged <13 years, the parent/guardian was asked to complete the record with input from the child as appropriate. Irrespective of age, however, we encouraged participants to get support from the main cook (e.g. mother and wife) when necessary. Within a few days after each recording day (usually the next day), the research dietitian collected the recording diary, checked the completeness of recording and recorded additional information if necessary. All the collected diaries were checked by trained dietitians at the central office in terms of coding, recorded weights and descriptions of items consumed. Data on dietary intake were not available in this study because the use of these data is not currently permitted by the Ministry of Health, Labour and Welfare, Japan.

### Definition and creation of eating pattern variables

The food diary sheet used was based on a typical Japanese eating pattern, which comprises breakfast, lunch, dinner and snacks, and these eating occasions were prescribed in the diary. Thus, the eating event (eating occasion) name used in the present analysis was based on this classification. Multiple entries of eating events into a section of breakfast, lunch or dinner were extremely rare in this study (only six cases); in these cases, the first eating event was considered the corresponding eating event (i.e. breakfast, lunch, or dinner), and the following eating events were considered snacks.

During the diet recording, participants were asked to report the clock time when a food or beverage was consumed (both start and finish times). Consequently, all items reported in an eating event were given the same clock time and event name in the food diary. Based on these data, several eating pattern variables were created mainly based on previous studies^(11–13,26)^ as described below. Unless otherwise indicated, the mean value over 8 d was used for each participant.

#### Eating frequency

In this study, eating occasions were defined as any separate intake occasion (with a discrete start clock time and name) except for eating occasions consisting of water only (tap and mineral water), which were excluded^(11)^. Thus, eating frequency was defined as the total number of eating occasions per day, which consist of foods only, drinks only or foods and drinks combined. Based on participant-identified name of eating occasions, eating frequency variables for meals (breakfast, lunch and dinner combined), snacks and all eating occasions were calculated for each participant.

#### Clock time for the start of eating

Based on information on start clock time of eating occasions, clock time for the start of eating breakfast, lunch, dinner and the first and the last eating occasions were created. For each individual, we calculated mean values over data-available days for clock time for the start of first and last eating occasions. We also calculated mean values on consumption days with data needed for clock time for the start of breakfast, lunch and dinner; participants who reported no consumption of these meals on any of 8 recording days had missing values on these variables.

#### Time spent on eating

Time spent on eating (breakfast, lunch, dinner and all snacks combined) was defined as time difference between the finish and start time of eating occasions. For these variables, we calculated mean values on consumption days with data needed, and participants who reported no consumption on any of 8 recording days had missing values on these variables.

#### Time between eating occasions

Time between eating occasions was defined as time between end of meal and start of next meal. For each day, the mean value of time between eating occasions was calculated, and then mean value over data-available days was calculated for each participant.

#### Length of ingestion period

Length of ingestion period was defined as time between the start clock time of the first eating occasion and the finish clock time of the last eating occasion. For each participant, we calculated mean value based on data-available days.

#### Variability of eating patterns

Variability in each eating pattern variable was calculated by adding the absolute difference between the mean value and that in each day divided by the number of days, with a higher value indicating a larger variability in eating patterns^(26)^. For each individual, mean daily values over 8 dietary recording days were used for eating frequency variables; mean values over data-available days for clock time for the start of first and last eating occasions, time between eating occasions, and length of ingestion period; mean values on consumption days with data needed for clock time for the start of breakfast, lunch and dinner and time spent on eating breakfast, lunch, dinner, and snacks. For variables based on breakfast, lunch, dinner and snacks, only participants who reported consumption of the corresponding eating episode on ≥ 4 d (with complete information) were included.

### Assessment of basic characteristics

Age at the time of start of the study was calculated based on birth date. Anthropometric measurements were performed by either family members or research dietitians using standard procedures. Body height (nearest 0·1 cm) and weight (nearest 0·1 kg) were measured, while the participants were barefoot and wearing light clothes only. When measurement was not available (*n* 23), self-reported (or parent-reported) height and weight were used. BMI (kg/m^2^) was calculated by the commonly used formula, namely weight (kg) divided by height squared (m^2^). Information on annual household income was collected using a question with possible sixteen categories, which were subsequently aggregated into three categories (< 4 million, ≥ 4 to < 7 million and ≥ 7 million Japanese yen). Information on education level and employment status was also collected for adult participants (aged 20–79 years), who were grouped into three categories for the former (junior high school or high school, college or vocational school and university or higher) and into four categories for the latter (full-time job, part-time job, student and unemployed).

### Analytic sample

For analysis, we excluded from the initial sample of 4268 participants 111 participants with < 8-d dietary record data, 102 participants who had 8-d dietary data but whose dietary assessment was conducted on 2 consecutive days at least in 1 season and seven participants who had nonconsecutive 8-d dietary data but whose dietary assessment was not conducted in appropriate months (i.e. October, November and December for fall, January, February and March for winter, April, May and June for spring and July, August and September for summer). After further excluding twelve participants who became pregnant during data collection and four participants who lived in a different region (which we recognised after the start of data collection), the final analysis sample comprised 4032 participants (see online supplementary material, Supplemental Figure S1). Further exclusion of participants whose dietary assessment was not conducted in accordance with the schedule assigned (*n* 324) did not alter the findings of the study (data not shown); therefore, these participants were retained in the analysis.

### Statistical analysis

All statistical analyses were performed for adults (aged 20–79 years) and children (aged 1–19 years) separately, using SAS statistical software (version 9.4, SAS Institute Inc.). Data are presented as means and sd for continuous variables and as the numbers and percentages of participants for categorical variables. First, the number and proportion of participants by number of reporting days of consumption of breakfast, lunch, dinner and snacks were calculated. Then, distribution of clock time for the start of eating breakfast, lunch, dinner and snacks was described. Finally, descriptive statistics on eating pattern variables was provided for the whole sample as well as by sex and age group (20–39, 40–59 and 60–79 years for adults and 1–6, 7–13 and 14–19 years for children). Differences in eating pattern variables between sex and across age categories were examined on the basis of independent *t* test and ANOVA, respectively. All reported *P* values are two-tailed, and *P* values <0·05 were considered statistically significant. When the overall *P* value from ANOVA was <0·05, a Bonferroni’s post hoc test was performed.

## Results

This analysis included 2681 adults aged 20–79 years (1325 men and 1356 women) and 1351 children aged 1–19 years (680 boys and 671 girls) who completed nonconsecutive 8-d dietary record over a year (Table 1). A total of 16·3 % of dietary record came from Monday, 15·6 % from Tuesday, 16·0 % from Wednesday, 15·0 % from Thursday, 13·9 % from Friday, 8·3 % from Saturday and 14·9 % from Sunday. The percentage of participants (adults and children combined) who reported consumption of breakfast, lunch and dinner on all the 8 dietary recording days was 88·1 %, 92·6 % and 95·9 %, respectively (see online supplementary material, Supplemental Table S1). In total, 81·4 % of participants reported consumption of all three main meals on all 8 d, with additional 12·6 % of participants reporting consumption of two of three main meals on all 8 d as well as that of the remaining meal on at least 4 d. In contrast, the prevalence of no consumption of each of these meals on all 8 d was very low (0·7 % for breakfast, 0·1 % for lunch and 0·05 % for dinner). For snacks, 54·9 % of participants reported consumption on all 8 d, with 4·1 % of them reporting no consumption on any of 8 d.

**Table 1:** Basic characteristics of study population

	Adults (aged 20–79 years)						Children (aged 1–19 years)					
	All (*n* 2681)		Male (*n* 1325)		Female (*n* 1356)		All (*n* 1351)		Male (*n* 680)		Female (*n* 671)	
	Mean	sd	Mean	sd	Mean	sd	Mean	sd	Mean	sd	Mean	sd
Age (years)	49·7	16·9	49·6	17·1	49·7	16·8	9·9	5·4	9·8	5·4	9·9	5·5
Body height (cm)	162·7	9·0	169·3	6·3	156·2	6·0	133·6	28·7	135·7	30·7	131·4	26·4
Body weight (kg)	61·2	12·2	68·0	11·2	54·6	9·2	34·6	18·2	36·1	20·0	33·1	16·0
BMI (kg/m^2^)	23·0	3·6	23·7	3·4	22·4	3·6	17·7	3·4	17·7	3·6	17·7	3·3
Annual household income (%)*												
<4 million Japanese yen	36·0		33·6		38·5		15·3		15·0		15·5	
≥4 to < 7 million Japanese yen	34·0		35·8		32·2		39·7		41·5		37·8	
≥7 million Japanese yen	30·0		30·6		29·3		45·1		43·5		46·7	
Education level (%)†												
Junior high school or high school	38·0		37·1		38·8		–		–		–	
College or technical school	30·7		20·0		41·2		–		–		–	
University or higher	31·3		42·8		20·0		–		–		–	
Employment status (%)‡												
Full-time job	66·6		72·8		60·6		–		–		–	
Part-time job	12·6		8·5		16·7		–		–		–	
Student	1·3		1·4		1·3		–		–		–	
Unemployed	19·4		17·2		21·5		–		–		–	
Diet recording group (%)												
8 weekdays	45·4		45·0		45·8		47·5		46·9		48·0	
4 weekdays and 4 weekend days	45·0		45·4		44·7		47·6		48·1		47·1	
Other combinations	9·6		9·7		9·5		5·0		5·0		4·9	

* *n* 2656 for adults and 1331 for children (because of missing information).

† Available for adults only (*n* 2664 because of missing information).

‡ Available for adults only.

**Fig. 1:**
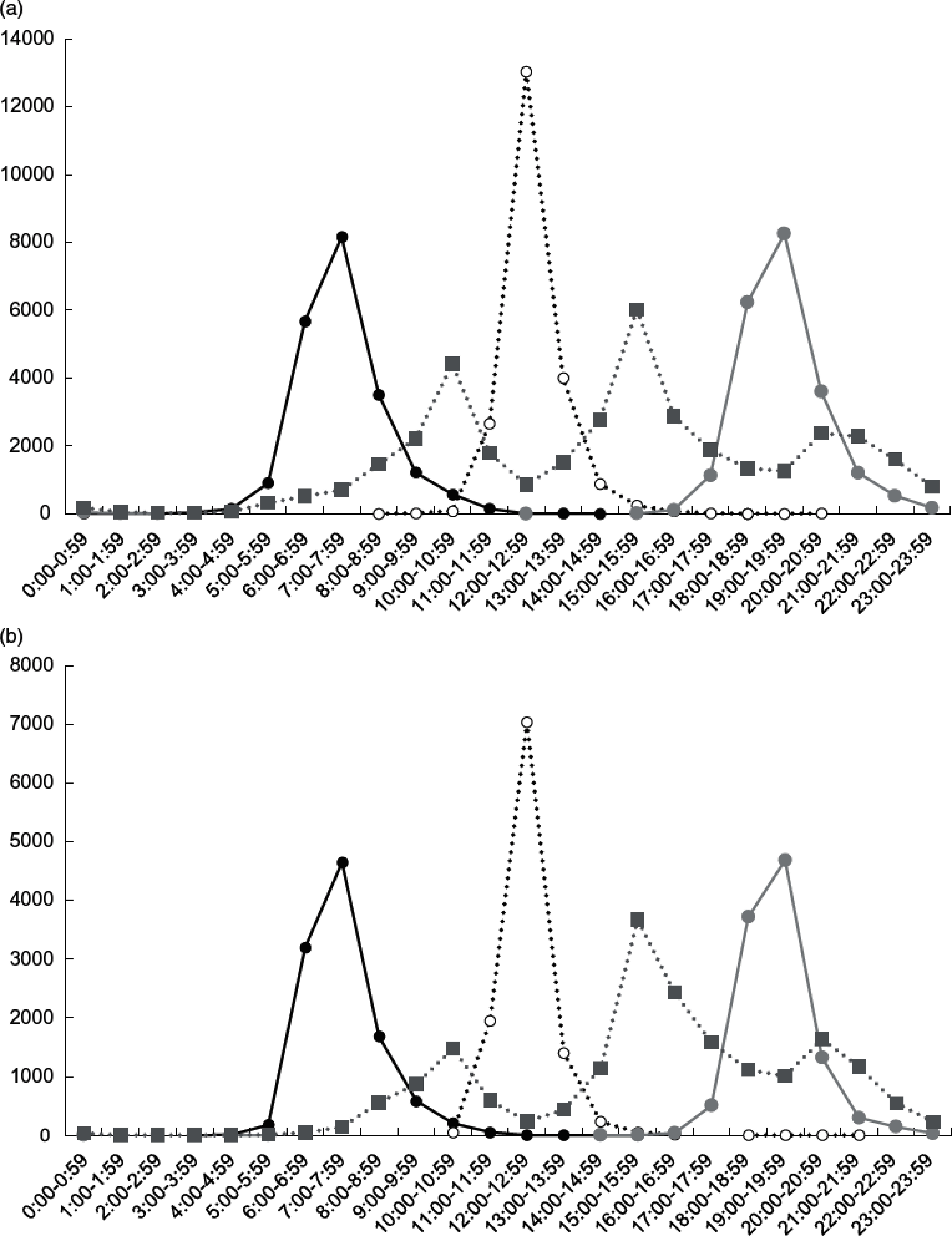
Distribution of clock time for the start of eating breakfast, lunch, dinner and snacks reported by 2681 Japanese adults aged 20–79 years (a) and by 1351 Japanese children aged 1–19 years (b). The total number of breakfast, lunch, dinner and snacks reported by adults in 8-d dietary record (with information on clock time) is 20 369, 20 982, 21 269 and 37 227, respectively. The corresponding number in children is 10 576, 10 732, 10 753 and 18 960, respectively. 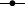 Breakfast; 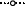
lunch; 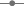
dinner; 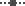
snacks

Figure 1 shows distribution of clock time for the start of breakfast, lunch, dinner and snacks reported by adults (a) and children (b) in 8-d dietary record. All three meals had a clear peak in timing (07.00–07.59 for breakfast, 12.00–12.59 for lunch and 19.00–19.59 for dinner). Within 3-h time slots with the central slot corresponding with the peak (i.e. 06.00–08.59 for breakfast, 11.00–13.59 for lunch and 18.00–20.59 for dinner) was reported a very large proportion of breakfast (85·1 % for adults and 90·1 % for children), lunch (93·8 % for adults and 96·8 % for children) and dinner (85·0 % for adults and 90·4 % for children). For the timing of snacks, there were three peaks in both adults and children (10.00–10.59, 15.00–15.59 and 20.00–20.59). Snacking after dinner was reported in 33 % of all dietary recording days.

For the population overall (*n* 4032), the mean value (sd) of eating frequency of meal, snacks and total eating occasions was 2·94 (0·19), 1·74 (1·18) and 4·68 (1·20) times/d, respectively. The mean (sd) clock time for the start of breakfast, lunch and dinner was 07.24 (00.48), 12.29 (00.31) and 19.15 (00.51) h, respectively. The mean (sd) time spent on breakfast, lunch, dinner and snacks was 19 (8), 25 (8), 34 (15) and 27 (34) min/d, respectively.

Tables 2 and 3 present descriptive data on eating pattern variables for adults and children, respectively, by sex and age group. Eating frequencies of meals, snacks and all eating occasions were higher in female adults (2·94, 1·85 and 4·79 times/d, respectively) than male adults (2·90, 1·62, and 4·52 times/d, respectively; all *P* < 0·0001), while eating frequencies of snacks and all eating occasions were higher in male children (1·83 and 4·80 times/d, respectively) than female children (1·68 and 4·65 times/d, respectively; both *P* ≤ 0·01). There was no sex difference in clock time for the start of eating, except for earlier start time for lunch in men (12.30 *v.* 12.34 h), earlier start time for dinner in women (19.16 *v.* 19.22 h) and earlier start time for the last eating occasion in girls (19.35 *v.* 19.46 h; all *P* ≤ 0·005). Longer time was spent on breakfast and lunch in both female adults (20 *v.* 18 min/d and 25 *v.* 23 min/d, respectively; both *P* < 0·0001) and female children (21 *v.* 19 min/d and 27 *v.* 25 min/d, respectively; both *P* ≤ 0·0006). Female adults also spent shorter time for dinner and snacks and had shorter time between eating occasions (34 *v.* 36 min/d, 25 *v.* 32 min/d and 3·2 *v.* 3·6 h, respectively; all *P* ≤ 0·0008). Female children also spent longer time for dinner and had shorter length of ingestion period (33 *v.* 31 min/d and 12·6 *v.* 12·8 h, respectively; both *P* ≤ 0·008). In terms of age, compared with adults aged 20–39 years (and to a lesser extent with adults aged 40–59 years), adults aged 60–79 years had higher eating frequencies of meals and snacks or all eating occasions, earlier clock time for the start of all meals as well as the first and last eating occasions, longer time spent on meals but shorter time on snacks, shorter time between eating occasions and shorter length of ingestion period. Similar characteristics were shared by children aged 1–6 years (compared with those aged 7–13 years and 14–19 years).

**Table 2:** Eating patterns of Japanese adults aged 20–79 years as assessed by eating frequency, clock time for the start of eating, time spent on eating, time between eating occasions and length of ingestion period, by sex and age group*

	All			Male			Female				Age 20–39 years			Age 40–59 years			Age 60–79 years			
	*n*	Mean	sd	*n*	Mean	sd	*n*	Mean	sd	*P* †	*n*	Mean	sd	*n*	Mean	sd	*n*	Mean	sd	*P* ‡
Eating frequency (times/d)																				
Meals§	2681	2·92	0·21	1325	2·90	0·25	1356	2·94	0·16	< 0·0001	878	2·85^a^	0·28	898	2·93^b^	0·21	905	2·98^c^	0·09	< 0·0001
Snacks	2681	1·74	1·24	1325	1·62	1·24	1356	1·85	1·23	< 0·0001	878	1·61^a^	1·31	898	1·81^b^	1·25	905	1·80^b^	1·15	0·0006
All eating occasions	2681	4·66	1·27	1325	4·52	1·28	1356	4·79	1·25	< 0·0001	878	4·45^a^	1·37	898	4·73^b^	1·27	905	4·78^b^	1·15	< 0·0001
Clock time for the start of eating (hh:mm)																				
Breakfast	2655	7:24	0:53	1304	7:23	0:55	1351	7:26	0:50	0·22	864	7:40^a^	0:57	886	7:16^b^	0:51	905	7:18^b^	0:46	< 0·0001
Lunch	2676	12:32	0:32	1320	12:30	0:33	1356	12:34	0:31	0·003	878	12:37^a^	0:37	894	12:34^a^	0:30	904	12:24^b^	0:28	< 0·0001
Dinner	2679	19:19	0:55	1323	19:22	0:59	1356	19:16	0:49	0·001	876	19:38^a^	0:54	898	19:31^b^	0:51	905	18:48^c^	0:45	< 0·0001
First eating occasion	2681	7:31	1:08	1325	7:33	1:14	1356	7:29	1:03	0·16	878	8:01^a^	1:21	898	7:19^b^	1:03	905	7:13^b^	0:50	< 0·0001
Last eating occasion	2681	19:57	1:16	1325	19:58	1:18	1356	19:56	1:13	0·52	878	20:17^a^	1:14	898	20:09^a^	1:13	905	19:25^b^	1:10	< 0·0001
Time spent on eating (min/d)																				
Breakfast	2655	19	9	1304	18	9	1351	20	8	< 0·0001	864	16^a^	7	886	18^b^	7	905	23^c^	9	< 0·0001
Lunch	2676	24	8	1320	23	8	1356	25	8	< 0·0001	878	23^a^	7	894	23^a^	8	904	26^b^	8	< 0·0001
Dinner	2679	35	17	1323	36	19	1356	34	15	0·0008	876	33^a^	15	898	36^b^	18	905	37^b^	17	< 0·0001
Snacks (total)||	2541	29	38	1230	32	43	1311	25	32	< 0·0001	827	36^a^	45	855	29^b^	42	859	21^c^	22	< 0·0001
Time between eating occasions (h)	2681	3·4	1·2	1325	3·6	1·3	1356	3·2	1·0	< 0·0001	878	3·6^a^	1·2	898	3·4^b^	1·2	905	3·1^c^	1·0	< 0·0001
Length of ingestion period (h)	2681	13·0	1·5	1325	13·0	1·5	1356	13·0	1·4	0·58	878	12·8^a^	1·6	898	13·4^b^	1·4	905	12·8^a^	1·2	< 0·0001

* For each individual, mean daily values over 8 dietary recording days were used for eating frequency variables; mean values over data-available days for clock time for the start of first and last eating occasions, time between eating occasions and length of ingestion period; mean values on consumption days with data needed for clock time for the start of breakfast, lunch and dinner and time spent on eating breakfast, lunch, dinner and snacks. For variables based on breakfast, lunch, dinner and snacks, participants who reported no consumption on any of 8 recording days were excluded (*n* 26, 5, 2 and 139, respectively).

† Sex difference examined based on independent *t* test.

‡ Age group difference examined based on ANOVA. When the overall *P* from ANOVA was < 0·05, a Bonferroni’s post hoc test was performed; values within each variable with unlike superscript letters are significantly different (*P* < 0·05).

§ Including breakfast, lunch and dinner.

|| One participant who reported consumption of snacks but provided no information on clock time was excluded.

**Table 3:** Eating patterns of Japanese children aged 1–19 years as assessed by eating frequency, clock time for the start of eating, time spent on eating, time between eating occasions and length of ingestion period, by sex and age group*

	All			Male			Female				Age 1–6 years			Age 7–13 years			Age 14–19 years			
	*n*	Mean	sd	*n*	Mean	sd	*n*	Mean	sd	*P* †	*n*	Mean	sd	*n*	Mean	sd	*n*	Mean	sd	*P* ‡
Eating frequency (times/d)																				
Meals§	1351	2·97	0·11	680	2·97	0·11	671	2·97	0·11	0·71	448	2·99^a^	0·05	461	2·99^a^	0·05	442	2·92^b^	0·17	< 0·0001
Snacks	1351	1·76	1·03	680	1·83	1·04	671	1·68	1·02	0·008	448	2·19^a^	1·00	461	1·62^b^	0·93	442	1·47^b^	1·03	< 0·0001
All eating occasions	1351	4·73	1·05	680	4·80	1·06	671	4·65	1·04	0·01	448	5·18^a^	1·00	461	4·61^b^	0·9	442	4·39^c^	1·05	< 0·0001
Clock time for the start of eating (hh:mm)																				
Breakfast	1349	7:24	0:39	679	7:24	0:39	670	7:24	0:39	0·97	448	7:30^a^	0:33	461	7:13^b^	0:29	440	7:31^a^	0:50	< 0·0001
Lunch	1351	12:24	0:27	680	12:24	0:27	671	12:25	0:26	0·45	448	12:04^a^	0:22	461	12:29^b^	0:15	442	12:40^c^	0:27	< 0·0001
Dinner	1351	19:07	0:43	680	19:08	0:44	671	19:06	0:42	0·30	448	18:47^a^	0:32	461	19:10^b^	0:36	442	19:33^c^	0:47	< 0·0001
First eating occasion	1351	7:29	0:48	680	7:29	0:48	671	7:29	0:48	0·90	448	7:29^a^	0:34	461	7:14^b^	0:32	442	7:44^c^	1:07	< 0·0001
Last eating occasion	1351	19:41	1:10	680	19:46	1:14	671	19:35	1:06	0·005	448	19:18^a^	1:03	461	19:32^b^	0:58	442	20:13^c^	1:18	< 0·0001
Time spent on eating (min/d)																				
Breakfast	1349	20	7	679	19	7	670	21	8	0·0006	448	25^a^	7	461	19^b^	6	440	15^c^	5	< 0·0001
Lunch	1351	26	7	680	25	7	671	27	7	0·0004	448	31^a^	7	461	25^b^	6	442	22^c^	6	< 0·0001
Dinner	1351	32	9	680	31	9	671	33	9	0·0003	448	35^a^	8	461	31^b^	9	442	29^c^	9	< 0·0001
Snacks (total)	1326	23	24	669	24	24	657	22	24	0·15	447	18^a^	15	453	27^b^	27	426	24^b^	28	< 0·0001
Time between eating occasions (h)	1351	3·2	0·9	680	3·1	0·9	671	3·2	0·9	0·13	448	2·6^a^	0·6	461	3·3^b^	0·9	442	3·7^c^	1·0	< 0·0001
Length of ingestion period (h)	1351	12·7	1·1	680	12·8	1·1	671	12·6	1·1	0·008	448	12·3^a^	0·8	461	12·8^b^	0·8	442	13·0^c^	1·4	< 0·0001

* For each individual, mean daily values over 8 dietary recording days were used for eating frequency variables; mean values over data-available days for clock time for the start of first and last eating occasions, time between eating occasions and length of ingestion period; mean values on consumption days with data needed for clock time for the start of breakfast, lunch and dinner and time spent on eating breakfast, lunch, dinner and snacks. For variables based on breakfast and snacks, participants who reported no consumption on any of 8 recording days were excluded (*n* 2 and 25, respectively).

† Sex difference examined based on independent *t* test.

‡ Age group difference examined based on ANOVA. When the overall *P* from ANOVA was < 0·05, a Bonferroni’s post hoc test was performed; values within each variable with unlike superscript letters are significantly different (*P* < 0·05).

§ Including breakfast, lunch and dinner.

Variability of eating patterns (calculated by adding the absolute difference between the mean value and that in each day divided by the number of days) is shown in Supplemental Table 2 (for adults) and Supplemental Table 3 (for children). On average, variability of meal frequency was small compared with that of snack frequency and total eating frequency. Both mean variability of clock time for the start of eating (< 1 h) and mean variability of time spent on meals (< 10 min/d) were also small. Conversely, mean variability of time spent on snacks was somewhat large (21·2 min/d for adults and 17·8 min/d for children).

Compared with male adults, female adults showed smaller variation in all eating pattern variables, except for no difference in snack frequency, total eating frequency, clock time for lunch and the last eating occasion and time spent on breakfast, as well as larger variability of time spent on lunch. For children, there was no sex difference, except for smaller variability of snack frequency and total eating frequency and larger variability of clock time for dinner and time spent on breakfast and dinner in females. In terms of age, adults aged 60–79 years had smaller variability of all eating pattern variables than younger adult groups, except for no difference in snack frequency. Smaller variability of eating patterns was also observed in children aged 1–6 years (compared with older child groups), except for no difference in variability of snack and total eating frequency and larger variability of time spent for breakfast and lunch.

## Discussion

To our knowledge, this is the first comprehensive report on patterning of eating behaviours under free-living conditions, particularly time spent on eating and variability of eating patterns. Eating frequency has been the most widely investigated variable of eating behaviours in adults. In the European Prospective Investigation into Cancer and Nutrition (EPIC) study consisting of ten European countries, mean total eating frequency (using the definition of eating occasion identical to that used in this study) varied across countries (4·9 to 7·0 times/d), with a trend for lower eating frequency in Mediterranean countries (Greece, Spain, Italy and France) compared with central European (Germany, the Netherlands and UK) and Nordic (Denmark, Sweden and Norway) countries^(11)^. While the definition of eating occasions is inconsistent across studies, mean total eating frequency in a national representative adult population has also been reported from the USA (5·0 times/d)^(13)^, Australia (ranging from 4·9 to 5·9 times/d, depending on sex and age group)^(12)^ and the UK (7·8 times/d for men and 7·6 times/d for women)^(14)^. In contrast, mean total eating frequency in the present Japanese adults (4·7 times/d), which was identical to that in a small previous Japanese study^(31)^, was consistently lower than that observed in Western countries. Given that daily meal frequency is generally close to three times in many studies^(11–13)^, this is clearly due to difference in snack frequency, with a lower mean in Japanese population (1·7 and 1·8 times/d in the present and previous^(31)^ studies, respectively) compared with Western populations (ranging from 2·1 to 4·2 times/d, depending on studies)^(11–13)^.

In contrast, fewer studies have attempted to characterise other potentially important features of eating patterns. It appears that meal skipping is rare in Japan (86 %, 91 % and 96 % of adults reporting consumption of breakfast, lunch and dinner, respectively, over all 8 d of dietary recording) compared with Western populations. In EPIC study, the proportion of consumers on a diet recall day ranged from 86·0 % to 99·8 % for breakfast, from 72·4 % to 100 % for lunch and from 89·2 % to 100 % for dinner^(11)^. The corresponding value in US adults from NHANES 2009–2014 was 85 %, 79 % and 93 %, respectively^(13)^. With regard to timing of eating, the mean clock time for the start of eating in US adults was 08.08 h for the first eating occasion, 08.11 h for breakfast, 12.43 h for lunch 18.24 h for dinner and 20.18 for the last eating occasion^(13)^. Compared with US population, our Japanese participants tended to consume earlier meals at earlier time (mean: 07.31 h for the first eating occasion, 07.25 h for breakfast and 12.32 h for lunch) but later meals at later time (19.19 h for dinner and 19.57 h for the last eating occasion). Consequently, the length of eating period was longer in the present Japanese (mean: 13.00 h) than the US adults (mean: 12.20 h)^(13)^. Nevertheless, time between eating occasions was longer in the present Japanese (mean: 3.40 h) than the US adults (mean: 2.50 h)^(13)^, mainly because of lower eating (snack) frequency in the former.

In this study, snacking after dinner was observed in only 33 % of total dietary recording days, which is a little more frequent compared with Mediterranean countries (21 % to 33 %) but much less frequent than other Western countries (49 % to 87 %)^(11,13)^. In a small study based on 16-d dietary record, the Japanese eating pattern was, on average, characterised by small snacks (11 % of total energy intake), as well as relatively large three main meals (percentage of total energy intake: 21 % for breakfast, 32 % for lunch and 40 % for dinner)^(32)^. A similar eating pattern (three main meals, with infrequent snacks particularly at night) was also observed in a representative sample of Taiwanese adults^(33)^. It would be of interest to clarify if this eating pattern is prevalent in East Asian countries with similar social and cultural backgrounds.

For time spent on eating, we are unaware of any previous studies which can be compared with the present findings. In the present population, a longer time was spent consuming later meals (mean: 19 min for breakfast, 24 min for lunch and 35 min for dinner), which is consistent with a previous observation in Japanese that a larger amount of energy was consumed in later meals (mean: 23 %, 30 % and 40 %, respectively)^(34)^. We are also unaware of previous studies investigating variability of eating patterns. In this population, it seemed that variability of eating patterns related to meals was relatively small compared with those for snacks.

Taken together, eating patterns of Japanese adults may be characterised by a relatively stable nature of frequency, timing and time spent with regard to meals (breakfast, lunch and dinner) as well as snacks which are less frequently consumed but exhibit greater variability in both timing and time spent. It seems that these are somewhat in common in eating patterns in Mediterranean regions characterised by less eating frequency^(12)^ and later consumption of meals and snacks^(18)^. As these characteristics may be favourably associated with health outcomes beyond food selection and nutrient composition^(9)^, further studies on the association of eating patterns with food and nutrient intake, diet quality and health outcomes would merit more exploration.

Eating patterns in children as well as association between eating patterns and basic characteristics have been poorly investigated. In this study, eating patterns in children were on average comparable with those in adults, which may be reasonable given that adults are generally responsible for children’s diet. For the associations of eating patterns with sex and age, the most consistent finding was that older adults and younger children have more stable and regular eating patterns with regard to frequency, timing and time spent on eating compared with younger adults and older children, respectively. This may reflect busy lifestyle in younger adults and older children. In any case, further studies on these topics are warranted.

The advantages of the present study include the use of actual eating behaviour data based on food diary with a large number of recording days (8 d) collected throughout a year, use of a wide range of eating pattern variables with clear definitions and a nationwide large sample. However, there are also several limitations. First, although the sampling was conducted so that regional difference in population proportion is reflected, the present population is not a nationally representative sample of general Japanese but rather volunteers. Given the burden required for dietary recording, it is conceivable that the participants were more representative of the health conscious nature. Nevertheless, distribution of annual household income in the present population was similar to that in a national representative sample (45·0 %, 26·9 % and 28·0 % for <4, ≥4 to <7 and ≥7 million Japanese yen, respectively, in all households; 19·3 %, 36·2 % and 44·5 %, respectively, in households with a child/children)^(35)^, although education level in the present adult population was somewhat high compared with a national representative sample (54·6 % for junior high school or high school, 20·8 % for college or vocational school and 24·6 % for university or higher)^(36)^. Further, mean (sd) values of body height, weight and BMI in our adult participants were also similar to those in a national representative sample aged ≥ 20 years (men: 167·6 (7·0) cm, 67·0 (11·5) kg and 23·8 (3·4) kg/m^2^, respectively; women: 154·1 (6·9) cm, 53·6 (9·4) kg and 22·6 (3·7) kg/m^2^, respectively)^(37)^. Thus, there may be no strong reason for considering that the present participants largely differ from general Japanese population.

Second, while all the dietary data were derived from dietary record, nature and extent of measurement error of self-reported (parent-reported) information on eating patterns are largely unknown. However, given that there are no objective markers of eating patterns examined here, we had no choice but relying on self-report on eating patterns. In this regard, we believe that dietary record is an optimal solution because we are able to collect a wide range of information (e.g. time) based on actual eating behaviours without relying on memory. Third, there is currently no consensus about what constitutes an eating occasion, a meal, a breakfast, a lunch, a dinner and a snack^(12,16,17)^. Although we used a widely used definition of eating occasions^(11)^ as well as meals and snacks^(11–13)^ to maximise comparability between studies, the present findings should be interpreted with these caveats in mind, and different results may be obtained if different definitions were applied. Finally, by design, dietary recording was conducted avoiding overnight working days (as well as days before and after these days). For overnight workers, dietary data might not fully represent their usual eating patterns because of a lack of data on overnight working days. Thus, further studies are needed to investigate eating patterns in overnight workers.

In conclusion, we provided comprehensive pictures of a range of eating pattern variables in a nationwide sample of Japanese. The major findings are as follows: meal skipping is quite rare in Japan; compared with Western populations, Japanese have on average a lower eating frequency (because of lower snack frequency); clock time for the start of breakfast and lunch is relatively early while that for lunch is relatively late; time spent on eating meals gradually increases during the day; variability of eating patterns is relatively small except for snacking and is smaller in older adults and younger children compared with younger adults and older children, respectively. The present findings serve as both a reference and an indication for future research on patterning of eating behaviours. The next step is to investigate associations of a variety of eating pattern variables with food and nutrient intake, diet quality and health outcomes.

## Supplementary material

For supplementary material accompanying this paper visit https://doi.org/10.1017/S1368980021000975. click here to view supplementary material

## References

[1] Kant AK (2004) Dietary patterns and health outcomes. J Am Diet Assoc 104, 615–635. 15054348 10.1016/j.jada.2004.01.010

[2] Hu FB (2002) Dietary pattern analysis: a new direction in nutritional epidemiology. Curr Opin Lipidol 13, 3–9. 11790957 10.1097/00041433-200202000-00002

[3] Newby PK & Tucker KL (2004) Empirically derived eating patterns using factor or cluster analysis: a review. Nutr Rev 62, 177–203. 15212319 10.1301/nr.2004.may.177-203

[4] Ax E , Warensjo Lemming E , Becker W et al. (2016) Dietary patterns in Swedish adults; results from a national dietary survey. Br J Nutr 115, 95–104. 26490112 10.1017/S0007114515004110

[5] Gazan R , Bechaux C , Crepet A et al. (2016) Dietary patterns in the French adult population: a study from the second French national cross-sectional dietary survey (INCA2) (2006–2007). Br J Nutr 116, 300–315. 27189191 10.1017/S0007114516001549 PMC4910537

[6] Hearty AP & Gibney MJ (2009) Comparison of cluster and principal component analysis techniques to derive dietary patterns in Irish adults. Br J Nutr 101, 598–608. 18577300 10.1017/S0007114508014128

[7] Kerver JM , Yang EJ , Obayashi S et al. (2006) Meal and snack patterns are associated with dietary intake of energy and nutrients in US adults. J Am Diet Assoc 106, 46–53. 16390666 10.1016/j.jada.2005.09.045

[8] Myhre JB , Loken EB , Wandel M et al. (2015) Meal types as sources for intakes of fruits, vegetables, fish and whole grains among Norwegian adults. Public Health Nutr 18, 2011–2021. 25384694 10.1017/S1368980014002481 PMC10271256

[9] Leech RM , Worsley A , Timperio A et al. (2015) Understanding meal patterns: definitions, methodology and impact on nutrient intake and diet quality. Nutr Res Rev 28, 1–21. 25790334 10.1017/S0954422414000262 PMC4501369

[10] Jacobs DR & Steffen LM (2003) Nutrients, foods, and dietary patterns as exposures in research: a framework for food synergy. Am J Clin Nutr 78, 508S–513S. 12936941 10.1093/ajcn/78.3.508S

[11] Huseinovic E , Winkvist A , Slimani N et al. (2016) Meal patterns across ten European countries – results from the European Prospective Investigation into Cancer and Nutrition (EPIC) calibration study. Public Health Nutr 19, 2769–2780. 27194183 10.1017/S1368980016001142 PMC10271196

[12] Leech RM , Worsley A , Timperio A et al. (2015) Characterizing eating patterns: a comparison of eating occasion definitions. Am J Clin Nutr 102, 1229–1237. 26447152 10.3945/ajcn.115.114660

[13] Kant AK (2018) Eating patterns of US adults: meals, snacks, and time of eating. Physiol Behav 193, 270–278. 29574043 10.1016/j.physbeh.2018.03.022

[14] Murakami K & Livingstone MB (2014) Eating frequency in relation to body mass index and waist circumference in British adults. Int J Obes 38, 1200–1206. 10.1038/ijo.2014.1 24406480

[15] Canuto R , da Silva Garcez A , Kac G et al. (2017) Eating frequency and weight and body composition: a systematic review of observational studies. Public Health Nutr 20, 2079–2095. 28578730 10.1017/S1368980017000994 PMC10261591

[16] Murakami K & Livingstone MB (2016) Associations between meal and snack frequency and overweight and abdominal obesity in US children and adolescents from National Health and Nutrition Examination Survey (NHANES) 2003–2012. Br J Nutr 115, 1819–1829. 27001436 10.1017/S0007114516000854

[17] Murakami K & Livingstone MB (2015) Eating frequency is positively associated with overweight and central obesity in US adults. J Nutr 145, 2715–2724. 26468490 10.3945/jn.115.219808

[18] Huseinovic E , Winkvist A , Freisling H et al. (2019) Timing of eating across ten European countries – results from the European Prospective Investigation into Cancer and Nutrition (EPIC) calibration study. Public Health Nutr 22, 324–335. 30326988 10.1017/S1368980018002288 PMC10260579

[19] Garaulet M & Gomez-Abellan P (2014) Timing of food intake and obesity: a novel association. Physiol Behav 134, 44–50. 24467926 10.1016/j.physbeh.2014.01.001

[20] Marinac CR , Sears DD , Natarajan L et al. (2015) Frequency and circadian timing of eating may influence biomarkers of inflammation and insulin resistance associated with breast cancer risk. PLoS One 10, e0136240. 26305095 10.1371/journal.pone.0136240 PMC4549297

[21] Almoosawi S , Vingeliene S , Karagounis LG et al. (2016) Chrono-nutrition: a review of current evidence from observational studies on global trends in time-of-day of energy intake, its association with obesity. Proc Nutr Soc 75, 487–500. 27327252 10.1017/S0029665116000306

[22] Farshchi HR , Taylor MA & Macdonald IA (2004) Regular meal frequency creates more appropriate insulin sensitivity and lipid profiles compared with irregular meal frequency in healthy lean women. Eur J Clin Nutr 58, 1071–1077. 15220950 10.1038/sj.ejcn.1601935

[23] Farshchi HR , Taylor MA & Macdonald IA (2004) Decreased thermic effect of food after an irregular compared with a regular meal pattern in healthy lean women. Int J Obes Relat Metab Disord 28, 653–660. 15085170 10.1038/sj.ijo.0802616

[24] Farshchi HR , Taylor MA & Macdonald IA (2005) Beneficial metabolic effects of regular meal frequency on dietary thermogenesis, insulin sensitivity, and fasting lipid profiles in healthy obese women. Am J Clin Nutr 81, 16–24. 15640455 10.1093/ajcn/81.1.16

[25] Sierra-Johnson J , Unden AL , Linestrand M et al. (2008) Eating meals irregularly: a novel environmental risk factor for the metabolic syndrome. Obesity 16, 1302–1307. 18388902 10.1038/oby.2008.203

[26] Murakami K & Livingstone MB (2015) Variability in eating frequency in relation to adiposity measures and blood lipid profiles in British children and adolescents: findings from the National Diet and Nutrition Survey. Int J Obes 39, 608–613. 10.1038/ijo.2015.7 25640770

[27] Shin A , Lim SY , Sung J et al. (2009) Dietary intake, eating habits, and metabolic syndrome in Korean men. J Am Diet Assoc 109, 633–640. 19328258 10.1016/j.jada.2008.12.015

[28] Robinson E , Almiron-Roig E , Rutters F et al. (2014) A systematic review and meta-analysis examining the effect of eating rate on energy intake and hunger. Am J Clin Nutr 100, 123–151. 24847856 10.3945/ajcn.113.081745

[29] Ohkuma T , Hirakawa Y , Nakamura U et al. (2015) Association between eating rate and obesity: a systematic review and meta-analysis. Int J Obes 39, 1589–1596. 10.1038/ijo.2015.96 26100137

[30] Statistics Bureau & Ministry of Internal Affairs and Communications (2015) Population and Households of Japan 2015. https://www.stat.go.jp/english/data/kokusei/2015/poj/mokuji.html (accessed July 2020).

[31] Murakami K , Shinozaki N , Livingstone MBE et al. (2020) Meal and snack frequency in relation to diet quality in Japanese adults: a cross-sectional study using different definitions of meals and snacks. Br J Nutr 124, 1219–1228. 32594916 10.1017/S0007114520002317 PMC7653514

[32] Murakami K , Shinozaki N , Livingstone MBE et al. (2020) Characterisation of breakfast, lunch, dinner, snacks in the Japanese context: an exploratory cross-sectional analysis. Public Health Nutr. Published online: 10 November 2020. doi: 10.1017/S1368980020004310. PMC9991704 33168120

[33] Chau CA , Pan WH & Chen HJ (2017) Employment status and temporal patterns of energy intake: nutrition and Health Survey in Taiwan, 2005–2008. Public Health Nutr 20, 3295–3303. 28931442 10.1017/S1368980017002476 PMC10261684

[34] Murakami K , Livingstone MBE & Sasaki S (2019) Meal-specific dietary patterns and their contribution to overall dietary patterns in the Japanese context: findings from the 2012 National Health and Nutrition Survey, Japan. Nutrition 59, 108–115. 30471523 10.1016/j.nut.2018.07.110

[35] Ministry of Health, Labour and Welfare (2017) Comprehensive Survey of Living Conditions 2017. https://www.mhlw.go.jp/toukei/saikin/hw/k-tyosa/k-tyosa17/dl/10.pdf (accessed July 2020).

[36] Statistics Bureau & Ministry of Internal Affairs and Communications, Japan (2018) Employment Status Survey, 2017. https://www.stat.go.jp/data/shugyou/2017/index2.html (accessed February 2021).

[37] Ministry of Health, Labour and Welfare (2018) National Health and Nutrition Survey 2017. https://www.mhlw.go.jp/stf/seisakunitsuite/bunya/kenkou_iryou/kenkou/eiyou/h29-houkoku.html (accessed July 2020).

