# Supplementary material for: Eating patterns in a nationwide sample of Japanese aged 1–79 years from MINNADE study: eating frequency, clock time for eating, time spent on eating and variability of eating patterns
Source: Public Health Nutr. 2021 Mar 5;25(6):1515–27. doi: 10.1017/S1368980021000975 (PMC9991801; doi:10.1017/S1368980021000975)
Supplement: Supplementary file 1 [file S1368980021000975sup001.docx]

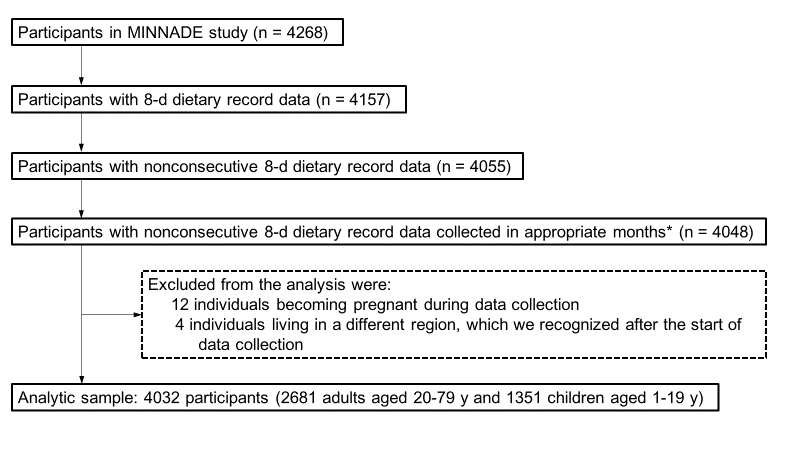


Supplemental Figure 1. Flow diagram of participants included in the present analysis. MINNADE, MINistry of health, labour and welfare-sponsored NAtionwide study on Dietary intake Evaluation. * In this study, 2-d dietary record was conducted in each season over 1-y period; appropriate months were considered October, November, and December for fall, January, February, and March for winter, April, May, and June for spring, and July, August, and September for summer.

Supplemental Table 1. Number and proportion of participants by number of reporting days of consumption of breakfast, lunch, dinner, and snacks in Japanese adults (aged 20-79 y) and children (aged 1-19 y)*

|  |  |  |  |  |  |  | Number of reporting days of consumption | | | | | |  |  |  |  |  |  |
| --- | --- | --- | --- | --- | --- | --- | --- | --- | --- | --- | --- | --- | --- | --- | --- | --- | --- | --- |
|  | 0 | | 1 | | 2 | | 3 | | 4 | | 5 | | 6 | | 7 | | 8 | |
|  | n | % | n | % | n | % | n | % | n | % | n | % | n | % | n | % | n | % |
| Adults (n 2681) |  |  |  |  |  |  |  |  |  |  |  |  |  |  |  |  |  |  |
| Breakfast | 26 | 1.0 | 17 | 0.6 | 14 | 0.5 | 25 | 0.9 | 33 | 1.2 | 41 | 1.5 | 73 | 2.7 | 140 | 5.2 | 2312 | 86.2 |
| Lunch | 5 | 0.2 | 3 | 0.1 | 4 | 0.2 | 8 | 0.3 | 9 | 0.3 | 21 | 0.8 | 43 | 1.6 | 156 | 5.8 | 2432 | 90.7 |
| Dinner | 2 | 0.1 | 0 | 0 | 0 | 0 | 2 | 0.1 | 7 | 0.3 | 3 | 0.1 | 11 | 0.4 | 93 | 3.5 | 2563 | 95.6 |
| Snacks | 139 | 5.2 | 83 | 3.1 | 92 | 3.4 | 98 | 3.7 | 127 | 4.7 | 154 | 5.7 | 235 | 8.8 | 389 | 14.5 | 1364 | 50.9 |
| Children (n 1351) |  |  |  |  |  |  |  |  |  |  |  |  |  |  |  |  |  |  |
| Breakfast | 2 | 0.2 | 1 | 0.1 | 4 | 0.3 | 3 | 0.2 | 9 | 0.7 | 9 | 0.7 | 25 | 1.9 | 57 | 4.2 | 1241 | 91.9 |
| Lunch | 0 | 0 | 0 | 0 | 0 | 0 | 0 | 0 | 1 | 0.1 | 0 | 0 | 14 | 1.0 | 36 | 2.7 | 1300 | 96.2 |
| Dinner | 0 | 0 | 0 | 0 | 0 | 0 | 0 | 0 | 0 | 0 | 0 | 0 | 8 | 0.6 | 38 | 2.8 | 1305 | 96.6 |
| Snacks | 25 | 1.9 | 20 | 1.5 | 27 | 2.0 | 37 | 2.7 | 49 | 3.6 | 64 | 4.7 | 100 | 7.4 | 179 | 13.3 | 850 | 62.9 |

For snacks, the number of days when at least one snack was reported is shown.

Supplemental Table 2. Variability of eating patterns of Japanese adults aged 20-79 y as assessed by eating frequency, clock time for the start of eating, time spent on eating, time between eating occasions, and length of ingestion period, by sex and age group*

|  |  | All |  |  | Male |  |  | Female |  |  | Age 20-39 y | | | Age 40-59 y | | | Age 60-79 y | | |  |
| --- | --- | --- | --- | --- | --- | --- | --- | --- | --- | --- | --- | --- | --- | --- | --- | --- | --- | --- | --- | --- |
|  | n | Mean | SD | n | Mean | SD | n | Mean | SD | P† | n | Mean | SD | n | Mean | SD | n | Mean | SD | P‡ |
| Eating frequency (times/d) |  |  |  |  |  |  |  |  |  |  |  |  |  |  |  |  |  |  |  |  |
| Meals§ | 2681 | 0.07 | 0.15 | 1325 | 0.08 | 0.16 | 1356 | 0.06 | 0.14 | 0.008 | 878 | 0.13^a^ | 0.19 | 898 | 0.06^b^ | 0.14 | 905 | 0.02^c^ | 0.08 | <0.0001 |
| Snacks | 2681 | 0.62 | 0.39 | 1325 | 0.60 | 0.41 | 1356 | 0.63 | 0.38 | 0.06 | 878 | 0.63 | 0.43 | 898 | 0.62 | 0.37 | 905 | 0.60 | 0.38 | 0.17 |
| All eating occasions | 2681 | 0.63 | 0.69 | 1325 | 0.62 | 0.41 | 1356 | 0.64 | 0.38 | 0.13 | 878 | 0.66^a^ | 0.42 | 898 | 0.63^ab^ | 0.37 | 905 | 0.60^b^ | 0.38 | 0.01 |
| Clock time for the start of eating (hh:mm) |  |  |  |  |  |  |  |  |  |  |  |  |  |  |  |  |  |  |  |  |
| Breakfast | 2597 | 0:48 | 1:19 | 1266 | 0:52 | 1:23 | 1331 | 0:43 | 1:14 | 0.003 | 814 | 1:19^a^ | 1:48 | 879 | 0:45^b^ | 1:11 | 904 | 0:22^c^ | 0:30 | <0.0001 |
| Lunch | 2661 | 0:40 | 1:18 | 1309 | 0:40 | 1:21 | 1352 | 0:39 | 1:15 | 0.74 | 872 | 0:55^a^ | 1:37 | 889 | 0:40^b^ | 1:21 | 900 | 0:23^c^ | 0:43 | <0.0001 |
| Dinner | 2677 | 0:43 | 1:27 | 1321 | 0:46 | 1:35 | 1356 | 0:39 | 1:18 | 0.02 | 876 | 1:01^a^ | 1:56 | 897 | 0:41^b^ | 1:19 | 904 | 0:25^c^ | 0:49 | <0.0001 |
| First eating occasion | 2681 | 0:37 | 0:35 | 1325 | 0:40 | 0:39 | 1356 | 0:34 | 0:31 | <0.0001 | 878 | 0:54^a^ | 0:43 | 898 | 0:35^b^ | 0:32 | 905 | 0:22^c^ | 0:20 | <0.0001 |
| Last eating occasion | 2681 | 0:59 | 1:20 | 1325 | 1:02 | 1:26 | 1356 | 0:56 | 1:14 | 0.053 | 878 | 1:16^a^ | 1:41 | 898 | 0:57^b^ | 1:16 | 905 | 0:44^c^ | 0:55 | <0.0001 |
| Time spent on eating (min/d) |  |  |  |  |  |  |  |  |  |  |  |  |  |  |  |  |  |  |  |  |
| Breakfast | 2597 | 5.1 | 5.1 | 1266 | 5.2 | 5.3 | 1331 | 5.0 | 4.9 | 0.35 | 814 | 5.9^a^ | 6.4 | 879 | 4.9^b^ | 4.9 | 904 | 4.5^b^ | 3.6 | <0.0001 |
| Lunch | 2661 | 6.5 | 5.6 | 1309 | 6.1 | 5.2 | 1352 | 6.9 | 5.8 | 0.0002 | 872 | 7.0^a^ | 5.7 | 889 | 6.5^ab^ | 5.9 | 900 | 5.9^b^ | 5.0 | 0.0001 |
| Dinner | 2677 | 9.1 | 9.5 | 1321 | 9.7 | 10.0 | 1356 | 8.5 | 9.1 | 0.0009 | 876 | 10.3^a^ | 11.3 | 897 | 9.5^a^ | 9.9 | 904 | 7.4^b^ | 6.7 | <0.0001 |
| Snacks (total) | 2269 | 21.2 | 29.1 | 1070 | 24.4 | 33.3 | 1199 | 18.3 | 24.4 | <0.0001 | 704 | 28.4^a^ | 36.9 | 779 | 21.1^b^ | 27.5 | 786 | 14.9^c^ | 19.9 | <0.0001 |
| Time between eating occasions (h) | 2681 | 0.67 | 0.41 | 1325 | 0.71 | 0.45 | 1356 | 0.62 | 0.37 | <0.0001 | 878 | 0.79^a^ | 0.46 | 898 | 0.68^b^ | 0.40 | 905 | 0.54^c^ | 0.32 | <0.0001 |
| Length of ingestion period (h) | 2681 | 1.35 | 1.88 | 1325 | 1.47 | 2.04 | 1356 | 1.24 | 1.70 | 0.001 | 878 | 1.90^a^ | 2.51 | 898 | 1.32^b^ | 1.70 | 905 | 0.85^c^ | 0.95 | <0.0001 |

* Variability in each eating pattern variable was calculated by adding the absolute difference between the mean value and that in each day divided by the number of days, with a higher value indicating a large variability in eating patterns. For each individual, mean daily values over 8 dietary recording days were used for eating frequency variables; mean values over data-available days for clock time for the start of first and last eating occasions, time between eating occasions, and length of ingestion period; mean values on consumption days with data needed for clock time for the start of breakfast, lunch, and dinner and time spent on eating breakfast, lunch, dinner, and snacks. For variables based on breakfast, lunch, dinner, and snacks, only participants who reported consumption of the corresponding eating episode on more than or equal to 4 days (with complete information) were included.

† Sex difference examined based on independent t-test.

‡ Age group difference examined based on ANOVA. When the overall P from ANOVA was <0.05, a Bonferroni’s post hoc test was performed; values within each variable with unlike superscript letters are significantly different (P <0.05).

§ Including breakfast, lunch, and dinner.

Supplemental Table 3. Variability of eating patterns of Japanese adults aged 1-19 y as assessed by eating frequency, clock time for the start of eating, time spent on eating, time between eating occasions, and length of ingestion period, by sex and age group*

|  |  | All |  |  | Male |  |  | Female |  |  | Age 1-6 y | | | Age 7-13 y | | | Age 14-19 y | | |  |
| --- | --- | --- | --- | --- | --- | --- | --- | --- | --- | --- | --- | --- | --- | --- | --- | --- | --- | --- | --- | --- |
|  | n | Mean | SD | n | Mean | SD | n | Mean | SD | P† | n | Mean | SD | n | Mean | SD | n | Mean | SD | P‡ |
| Eating frequency (times/d) |  |  |  |  |  |  |  |  |  |  |  |  |  |  |  |  |  |  |  |  |
| Meals§ | 1351 | 0.04 | 0.11 | 680 | 0.04 | 0.11 | 671 | 0.04 | 0.11 | 0.49 | 448 | 0.02^a^ | 0.07 | 461 | 0.01^a^ | 0.07 | 442 | 0.09^b^ | 0.16 | <0.0001 |
| Snacks | 1351 | 0.58 | 0.33 | 680 | 0.61 | 0.33 | 671 | 0.56 | 0.33 | 0.004 | 448 | 0.59 | 0.34 | 461 | 0.58 | 0.33 | 442 | 0.58 | 0.32 | 0.85 |
| All eating occasions | 1351 | 0.59 | 0.33 | 680 | 0.62 | 0.33 | 671 | 0.56 | 0.33 | 0.004 | 448 | 0.59 | 0.34 | 461 | 0.59 | 0.33 | 442 | 0.59 | 0.32 | 0.94 |
| Clock time for the start of eating (hh:mm) |  |  |  |  |  |  |  |  |  |  |  |  |  |  |  |  |  |  |  |  |
| Breakfast | 1342 | 0:42 | 1:01 | 675 | 0:42 | 1:00 | 667 | 0:42 | 1:01 | 0.95 | 448 | 0:24^a^ | 0:27 | 460 | 0:31^a^ | 0:27 | 434 | 1:11^b^ | 1:32 | <0.0001 |
| Lunch | 1351 | 0:27 | 0:45 | 680 | 0:28 | 0:50 | 671 | 0:27 | 0:39 | 0.68 | 448 | 0:23^a^ | 0:30 | 461 | 0:20^a^ | 0:27 | 442 | 0:40^b^ | 1:05 | <0.0001 |
| Dinner | 1351 | 0:34 | 0:50 | 680 | 0:31 | 0:40 | 671 | 0:37 | 0:58 | 0.03 | 448 | 0:28^a^ | 0:45 | 461 | 0:24^a^ | 0:22 | 442 | 0:49^b^ | 1:08 | <0.0001 |
| First eating occasion | 1351 | 0:35 | 0:29 | 680 | 0:34 | 0:29 | 671 | 0:36 | 0:30 | 0.29 | 448 | 0:23^a^ | 0:15 | 461 | 0:29^b^ | 0:21 | 442 | 0:53^c^ | 0:38 | <0.0001 |
| Last eating occasion | 1351 | 0:52 | 1:09 | 680 | 0:53 | 1:12 | 671 | 0:52 | 1:06 | 0.74 | 448 | 0:42^a^ | 0:53 | 461 | 0:43^a^ | 0:56 | 442 | 1:12^b^ | 1:28 | <0.0001 |
| Time spent on eating (min/d) |  |  |  |  |  |  |  |  |  |  |  |  |  |  |  |  |  |  |  |  |
| Breakfast | 1342 | 4.6 | 3.0 | 675 | 4.4 | 2.7 | 667 | 4.8 | 3.3 | 0.03 | 448 | 5.1^a^ | 3.3 | 460 | 4.0^b^ | 2.2 | 434 | 4.6^c^ | 3.3 | <0.0001 |
| Lunch | 1351 | 6.0 | 4.5 | 680 | 5.8 | 4.6 | 671 | 6.2 | 4.3 | 0.15 | 448 | 6.0 | 4.0 | 461 | 5.7 | 3.9 | 442 | 6.2 | 5.4 | 0.23 |
| Dinner | 1351 | 6.9 | 4.8 | 680 | 6.6 | 4.5 | 671 | 7.3 | 5.0 | 0.008 | 448 | 7.0^ab^ | 4.3 | 461 | 6.4^a^ | 4.4 | 442 | 7.3^b^ | 5.5 | 0.007 |
| Snacks (total) | 1242 | 17.8 | 24.9 | 630 | 18.1 | 22.6 | 612 | 17.5 | 27.0 | 0.66 | 444 | 11.6^a^ | 14.6 | 423 | 21.4^b^ | 25.9 | 375 | 21.3^b^ | 31.1 | <0.0001 |
| Time between eating occasions (h) | 1351 | 0.58 | 0.33 | 680 | 0.58 | 0.33 | 671 | 0.59 | 0.33 | 0.74 | 448 | 0.42^a^ | 0.25 | 461 | 0.61^b^ | 0.28 | 442 | 0.73^c^ | 0.36 | <0.0001 |
| Length of ingestion period (h) | 1351 | 1.06 | 1.26 | 680 | 1.08 | 1.45 | 671 | 1.03 | 1.04 | 0.52 | 448 | 0.72^a^ | 0.68 | 461 | 0.84^a^ | 0.73 | 442 | 1.63^b^ | 1.84 | <0.0001 |

* Variability in each eating pattern variable was calculated by adding the absolute difference between the mean value and that in each day divided by the number of days, with a higher value indicating a large variability in eating patterns. For each individual, mean daily values over 8 dietary recording days were used for eating frequency variables; mean values over data-available days for clock time for the start of first and last eating occasions, time between eating occasions, and length of ingestion period; mean values on consumption days with data needed for clock time for the start of breakfast, lunch, and dinner and time spent on eating breakfast, lunch, dinner, and snacks. For variables based on breakfast, lunch, dinner, and snacks, only participants who reported consumption of the corresponding eating episode on more than or equal to 4 days (with complete information) were included.

† Sex difference examined based on independent t-test.

‡ Age group difference examined based on ANOVA. When the overall P from ANOVA was <0.05, a Bonferroni’s post hoc test was performed; values within each variable with unlike superscript letters are significantly different (P <0.05).

§ Including breakfast, lunch, and dinner.
